# Sex matters in the association between cardiovascular health and incident dementia: evidence from real world data

**DOI:** 10.1186/s13195-024-01406-x

**Published:** 2024-03-14

**Authors:** Anna Ponjoan, Jordi Blanch, Ester Fages-Masmiquel, Ruth Martí-Lluch, Lia Alves-Cabratosa, María del Mar Garcia-Gil, Gina Domínguez-Armengol, Francesc Ribas-Aulinas, Lluís Zacarías-Pons, Rafel Ramos

**Affiliations:** 1 Vascular Health Research Group (ISV-Girona), Fundació Institut Universitari per a la Recerca a l’Atenció Primària de Salut Jordi Gol i Gurina (IDIAPJGol), C/Maluquer Salvador nº11, Girona, Catalonia 17002 Spain; 2 Girona Biomedical Research Institute (IDIBGI), Dr. Trueta University Hospital. Parc Hospitalari Martí I Julià, (Ed. M2), C/Dr. Castany S/N, Salt (Girona), Catalonia 17190 Spain; 3 Network for Research On Chronicity, Primary Care, and Health Promotion (RICAPPS), C/ Maluquer Salvador nº11, Girona, Catalonia 17002 Spain; 4 Atenció Primària, Gerència Territorial de Girona, Institut Català de la Salut. C/Mossèn Joan Pons S/N, Girona, 17001 Spain; 5 Translab Research Group, Department of Medical Sciences, University of Girona, C/Emili Grahit, 77, Girona, Catalonia 17071 Spain

**Keywords:** Electronic health records, General practitioner, Primary health care, Family practice, Heart disease risk factors, Diabetes, Hypertension, Blood pressure, Smoking, Gender

## Abstract

**Background:**

Cardiovascular health has been associated with dementia onset, but little is known about the variation of such association by sex and age considering dementia subtypes. We assessed the role of sex and age in the association between cardiovascular risk and the onset of all-cause dementia, Alzheimer’s disease, and vascular dementia in people aged 50–74 years.

**Methods:**

This is a retrospective cohort study covering 922.973 Catalans who attended the primary care services of the Catalan Health Institute (Spain). Data were obtained from the System for the Development of Research in Primary Care (SIDIAP database). Exposure was the cardiovascular risk (CVR) at baseline categorized into four levels of Framingham-REGICOR score (FRS): low (FRS < 5%), low-intermediate (5% ≤ FRS < 7.5%), high-intermediate (7.5% ≤ FRS < 10%), high (FRS ≥ 10%), and one group with previous vascular disease. Cases of all-cause dementia and Alzheimer’s disease were identified using validated algorithms, and cases of vascular dementia were identified by diagnostic codes. We fitted stratified Cox models using age parametrized as b-Spline.

**Results:**

A total of 51,454 incident cases of all-cause dementia were recorded over a mean follow-up of 12.7 years. The hazard ratios in the low-intermediate and high FRS groups were 1.12 (95% confidence interval: 1.08–1.15) and 1.55 (1.50–1.60) for all-cause dementia; 1.07 (1.03–1.11) and 1.17 (1.11–1.24) for Alzheimer’s disease; and 1.34 (1.21–1.50) and 1.90 (1.67–2.16) for vascular dementia. These associations were stronger in women and in midlife compared to later life in all dementia types. Women with a high Framingham-REGICOR score presented a similar risk of developing dementia — of any type — to women who had previous vascular disease, and at age 50–55, they showed three times higher risk of developing dementia risk compared to the lowest Framingham-REGICOR group.

**Conclusions:**

We found a dose‒response association between the Framingham-REGICOR score and the onset of all dementia types. Poor cardiovascular health in midlife increased the onset of all dementia types later in life, especially in women.

**Supplementary Information:**

The online version contains supplementary material available at 10.1186/s13195-024-01406-x.

## Background

Patients with cardiovascular disease are at higher risk of developing dementia [1, 2]. Emerging evidence suggests that the control of cardiovascular risk could help prevent dementia even in persons without cardiovascular disease [3]. Some validated cardiovascular risk scores (i.e., Framingham or Life Simple 7) have been associated with the development of all-cause dementia [4–6]; but little is known about this association considering dementia subtypes [4].

Age and sex are risk factors for both cardiovascular diseases and dementia; hence, they may play a role in the association between these two conditions. Regarding age, some studies reported significant associations between cardiovascular risk scores and incident dementia in both midlife and late life [7, 8], but other analyses found significant associations only in midlife [6]. Thus, the role of age in the association between cardiovascular risk and dementia remains unclear, as do the variations of such influence by sex, which are of particular interest.

Few studies have provided analyses by sex and they reported inconsistent findings on the association between cardiovascular risk score and cognitive function or dementia: the Whitehall II study reported a stronger association in men [9] and the SALSA study in women [10]; the Rush Memory and Aging Project found similar results between sexes [4]. The Framingham Heart Study assessed the role of both sex and age in the relationship between individual cardiovascular risk factors and onset of overall dementia. Nevertheless, the small sample size precluded consistent conclusions, and the authors recommended further investigation on the role of sex in larger cohorts [6]. Large sample sizes for the study of dementia can be obtained from clinical databases of electronic health records routinely collected in primary care which have been reported to be accurate in identifying dementia cases [11, 12].

We used data from a large primary care database to assess the role of sex and age in the association between cardiovascular risk and the onset of all-cause dementia, Alzheimer’s disease, and vascular dementia.

## Methods

We designed a retrospective cohort study that included all dementia-free individuals registered in the System for the Development of Research in Primary Care (SIDIAP) aged 50 to 74 years who attended the primary care services up to one year prior to the entry date (further details on exclusion criteria in Box S1). The SIDIAP is a valid and reliable database that contains longitudinal health records from about 6 million persons who attended the primary care services of the Catalan Health Institute (12% of the Spanish population and 80% of the Catalan population) [13]. The SIDIAP database contains comprehensive demographic information, clinical diagnoses (coded with the International Classification of Diseases, tenth revision, clinical modification (ICD-10-CM)), treatments (coded with the Anatomical Therapeutic Chemical Classification code, ATC), referrals, and hospitalizations. The SIDIAP data are representative of the Catalan population [14], and the records on cardiovascular risk factors and diseases [15], dementia [12], and Alzheimer’s disease [16] have been validated.

The recruitment period was from January 1 until December 31, 2008. The entry date was the first day of the participants’ month of birth. Follow-up lasted until the earliest of the following events: dementia, death, moving out to a region that was not covered by SIDIAP, or end of the study period — December 31, 2022.

The exposure was the level of cardiovascular risk at baseline. Patients with previous vascular diseases were included in the highest risk category (Box S2). Participants free of cardiovascular disease at baseline were classified in four groups according to their Framingham-REGICOR score (Fig. S1): low (score < 5%), low-intermediate (5% ≤ score < 7.5%), high-intermediate (7.5% ≤ score < 10%), and high (score ≥ 10%). The Framingham-REGICOR is the Spanish-calibrated adaptation of the Framingham score; it is valid and reliable for the population without previous cardiovascular disease aged between 35 and 74 years [17]. The Framingham-REGICOR score combines several variables (age, sex, smoking, type-2 diabetes mellitus (DM2), total cholesterol, high-density lipoprotein cholesterol (HDL), systolic and diastolic blood pressure) to estimate the 10-year risk of developing a fatal or non-fatal myocardial infarction, silent myocardial infarction, or angina pectoris [17].

The outcomes were incident cases of all-cause dementia, Alzheimer’s disease — both identified by validated algorithms [12, 16] — and of vascular dementia identified using diagnostic codes (Box S3). We considered the following covariates at baseline: age; sex; rurality; (rural/urban); measurements of weight, height, systolic and diastolic blood pressure, total cholesterol and HDL, triglycerides; diagnosis of DM2, hyperthyroidism, alcohol-related disorders, Parkinson’s disease, and depression; smoking (smoker/non-smoker) and obesity (obese/non-obese) (Box S4).

### Statistical analyses

Continuous and categorical variables were described with the mean (standard deviation, SD) and counts (percentages, %), respectively. We used 10 multiple imputations by chained equations [18] to replace the missing baseline values of total cholesterol, HDL, triglycerides, blood pressure, weight, and height (Box S5). We estimated the crude incidences for each type of dementia and their 95% confidence intervals (95% CI) assuming a Poisson distribution. Estimates were disaggregated by risk group, sex, or age where appropriate.

We used stratified Cox survival models that allowed us to assess predictors (age or Framingham-REGICOR) even if they did not satisfy the proportional hazard assumptions by adjusting for the baseline hazard function of each stratum (in our study, age groups: 50–54, 55–59, 60–64, 65–69, 70–74 years) [19]. First, we fitted stratified Cox models by age and sex, and considered follow-up as an underlying timescale. We built one model for each outcome adjusted by obesity [20], alcohol related disorders [21], rurality [22], hyperthyroidism [23], and depression [24]. The interaction between the Framingham-REGICOR score and sex was tested.

Then, we replicated the stratified Cox models including an interaction between the Framingham-REGICOR score, sex, and age to estimate the hazard ratios (HR) for the dementia outcomes per year of age; age was parameterized as a b-Spline [25]. Interactions between age, sex, and Framingham-REGICOR score were assessed using likelihood ratio tests. In all models, we considered the minimum baseline risk group as reference.

As sensitivity analyses, we calculated unadjusted models; we also replicated models using the case-complete dataset and used the imputed dataset with censoring at the first-ever dementia or cardiovascular disease record during follow-up. Finally, we calculated Fine-Gray multivariate models to assess a potential competing risk with death. Statistical significance was set at *p* < 0.05. All analyses were performed using the R software, version 4.3.0, and we used the MICE package for multiple imputation [26, 27].

## Results

We identified 1,200,700 persons and 922,973 of them fulfilled the inclusion criteria (Fig. S1). The average follow-up time was 12.7 years (SD = 3.1). A comparison of the complete cases and imputed datasets showed similar mean values regarding the imputed variables (Table S1).

The study participants were mainly women (59.9%), from urban areas, with a mean age of 61.7 years. The baseline characteristics and exposure groups, overall and by sex, are described in Table S2. The Framingham-REGICOR estimates were higher in men (Table S2). The low and low-intermediate risk groups included 92.3% of women and 69.1% of men; whereas the high-intermediate and high-risk groups included 3.6% of women and 19.5% of men (Table 1). Women presented worse lipid profile, higher blood pressure levels, and higher prevalence of hypertension, DM2, and obesity compared to men in all the Framingham-REGICOR groups (Table 1). Only smoking and alcohol-related disorders were more prevalent in men (Table 1). The age increment with the increase of Framingham-REGICOR risk was more pronounced in men: the mean age between the low and the high exposure groups increased 8.9 years in men, and 2.5 years in women (Table 1).

**Table 1:** Baseline cardiovascular risk factors of the study population by cardiovascular risk groups and sex

**Variable**	**Sex**^**a**^	**Exposure groups**^**b**^ (risk at baseline) *n* (%); mean [sd]				
		**Low CVR**	**Low-intermediate CVR**	**High-intermediate CVR**	**High CVR**	**Previous vascular disease**
*N*	W	462,824 (83.7)	47,482 (8.6)	13,462 (2.4)	6863 (1.2)	22,415 (4.1)
	M	178,757 (48.3)	77,084 (20.8)	38,536 (10.4)	33,745 (9.1)	41,804 (11.3)
Age (year)	W	61.3 [7.2]	63.4 [6.0]	63.8 [5.8]	63.8 [5.3]	66.1 [6.3]
	M	58.5 [6.3]	63.2 [6.5]	65.5 [6.2]	67.4 [5.5]	65.0 [6.5]
Total cholesterol (mg/dl)	W	216.5 [34.6]	218.1 [39.0]	216.1 [41.6]	218.9 [41.8]	200.5 [38.9]
	M	200.5 [35.6]	210.7 [36.0]	214.6 [35.8]	218.7 [37.7]	182.6 [38.9]
HDL (mg/dl)	W	64.2 [14.1]	48.9 [10.0]	45.1 [8.7]	40.9 [7.8]	56.9 [14.3]
	M	54.7 [13.2]	49.7 [11.7]	47.5 [10.5]	44.5 [9.5]	48.1 [12.3]
Systolic blood pressure (mmHg)	W	128.8 [14.1]	142.1 [15.6]	144.9 [16.1]	153.1 [17.3]	133.7 [16.8]
	M	128.8 [12.8]	136.1 [14.4]	140.6 [15.1)	145.8 [15.5]	133.7 [16.6]
Hypertension	W	154,958 (33.5)	27,982 (58.9)	9310 (69.2)	5210 (75.9)	15,169 (67.7)
	M	50,561 (28.3)	32,211 (41.8)	18,556 (48.2)	18,845 (55.8)	25,215 (60.3)
Smoking	W	32,211 (7.0)	5458 (11.5)	1527 (11.3)	941 (13.7)	1260 (5.6)
	M	23,170 (13.0)	20,713 (26.9)	13,854 (36.0)	18,432 (54.6)	9032 (21.6)
Diabetes mellitus, type 2	W	23,583 (5.1)	19,448 (41.0)	10,344 (76.8)	6229 (90.8)	6710 (29.9)
	M	17,087 (9.6)	10,198 (13.2)	5758 (14.9)	6036 (17.9)	6990 (16.7)
Alcohol-related disorders	W	2269 (0.5)	300 (0.6)	108 (0.8)	58 (0.9)	172 (0.8)
	M	6427 (3.6)	3038 (3.9)	1567 (4.1)	1499 (4.4)	2024 (4.8)
Body mass index (kg/m^2^)	W	28.6 (5.5)	30.8 (6.0)	32.1 (6.2)	32.8 (6.4)	30.5 (5.9)
	M	28.1 (4.7)	28.6 (4.8)	28.8 (4.8)	29.2 (4.9)	29.1 (4.8)

^a^Sex: M, men; W, women

^b^Exposure groups based on cardiovascular risk (CVR) at baseline: low (Framingham-REGICOR < 5%), low-intermediate (5% ≤ Framingham-REGICOR < 7.5%), high-intermediate (7.5% ≤ Framingham-REGICOR < 10%), high (Framingham-REGICOR ≥ 10%), and patients with history of vascular disease before the baseline

During follow-up, we identified 51,454 cases of all-cause dementia, 32,440 of Alzheimer’s disease, and 4716 of vascular dementia. The crude incidence rates were 4.46/1,000 person-years (95% CI: 4.45–4.47) for all-cause dementia, 2.79/1000 person-years (95% CI: 2.78–2.81) for Alzheimer’s disease, and 0.40/1000 person-years (95% CI: 0.39–0.41) for vascular dementia. Within each age category, the crude incidence rates tended to increase with the baseline cardiovascular risk, and these increments tended to be more evident in women than in men for all-cause dementia (Table 2) and subtypes (Tables S3–S4).

**Table 2:** Crude incidences (95% CI) of overall dementia disaggregated by sex, age, and exposure groups

**Sex**	**Age**	**Whole population**	Exposure groups* (risk at baseline) *n* (%); mean [sd]				
			**Low CVR**	**Low-intermediate CVR**	**High-intermediate CVR**	**High CVR**	**Previous vascular disease**
Women	50,55	0.39 (0.36–0.43)	0.37 (0.34–0.40)	0.62 (0.44–0.88)	1.17 (0.68–2.01)	1.70 (0.81–3.56)	0.96 (0.60–1.54)
	55,60	1.08 (1.03–1.14)	1.00 (0.94–1.05)	1.44 (1.24–1.67)	1.79 (1.38–2.33)	2.33 (1.66–3.26)	2.28 (1.81–2.89)
	60,65	3.16 (3.07–3.24)	2.93 (2.84–3.02)	3.62 (3.35–3.92)	4.15 (3.62–4.75)	4.90 (4.14–5.80)	5.33 (4.74–5.99)
	65,70	7.22 (7.07–7.37)	6.87 (6.71–7.03)	7.77 (7.32–8.23)	8.86 (8.03–9.78)	8.28 (7.19–9.54)	9.88 (9.14–10.68)
	70,75	14.51 (14.29–14.73)	14.10 (13.87–14.34)	14.79 (14.06—15.55)	15.06 (13.68–16.59)	16.27 (14.10–18.77)	18.15 (17.29–19.06)
	All	4.79 (4.74–4.84)	4.37 (4.32–4.42)	5.86 (5.65–6.08)	6.51 (6.10–6.94)	6.92 (6.34–7.57)	10.30 (9.91–10.70)
Men	50,55	0.45 (0.41–0.49)	0.41 (0.37–0.46)	0.52 (0.41–0.67)	0.40 (0.23–0.71)	0.62 (0.30–1.30)	0.80 (0.57–1.13)
	55,60	1.07 (1.01–1.14)	0.93 (0.86–1.01)	1.05 (0.91–1.22)	1.10 (0.87–1.40)	1.64 (1.26–2.14)	2.11 (1.79–2.49)
	60,65	2.79 (2.68–2.89)	2.44 (2.30–2.58)	2.80 (2.60–3.02)	2.96 (2.65–3.31)	3.05 (2.68–3.48)	3.96 (3.60–4.36)
	65,70	5.84 (5.68–6.01)	5.37 (5.10–5.66)	5.37 (5.07–5.69)	5.51 (5.11–5.95)	6.39 (5.93–6.89)	7.70 (7.20–8.23)
	70,75	11.88 (11.64–12.14)	11.39 (10.83–11.98)	10.99 (10.51–11.49)	11.15 (10.61–11.73)	11.71 (11.19–12.26)	14.72 (14.05–15.41)
	All	3.95 (3.89–4.01)	2.22 (2.15–2.29)	4.29 (4.13–4.46)	5.72 (5.50–5.96)	7.33 (7.05–7.61)	7.27 (7.03–7.52)

^*^Exposure groups based on cardiovascular risk (CVR) at baseline: low (Framingham-REGICOR < 5%), low-intermediate (5% ≤ Framingham-REGICOR < 7.5%), high-intermediate (7.5% ≤ Framingham-REGICOR < 10%), high (Framingham-REGICOR ≥ 10%), and patients with history of vascular disease before the baseline

Stratified Cox models showed a dose–response association between cardiovascular risk and dementia onset: the higher the categorized Framingham-REGICOR risk at baseline, the higher the risk of developing all-cause dementia, Alzheimer’s disease, and vascular dementia (Table 3). The interactions between the Framingham-REGICOR score and sex were significant for all-cause dementia (*p* < 0.001) and vascular dementia (*p* = 0.02) but not for Alzheimer’s disease (*p* = 0.25). Disaggregated results by sex are shown in Table 3.

**Table 3:** Hazard ratios (95% CI) of the exposure groups obtained in the stratified Cox models for the overall population and by sex

Population	Exposure groups	All-cause dementia	Alzheimer’s disease	Vascular dementia
Overall	Low CVR	Reference	Reference	Reference
	Low-intermediate CVR	1.12 (1.08–1.15)	1.07 (1.03–1.11)	1.34 (1.21–1.50)
	High-intermediate CVR	1.17 (1.12–1.21)	1.10 (1.04–1.16)	1.52 (1.35–1.71)
	High CVR	1.29 (1.24–1.35)	1.17 (1.11–1.24)	1.90 (1.67–2.16)
	Previous CVD	1.55 (1.50–1.60)	1.17 (1.13–1.22)	3.68 (3.39–3.99)
Women	Low CVR	Reference	Reference	Reference
	Low-intermediate CVR	1.14 (1.10–1.19)	1.06 (1.01–1.12)	1.47 (1.28–1.70)
	High-intermediate CVR	1.21 (1.13–1.29)	1.09 (1.00–1.18)	1.70 (1.37–2.11)
	High CVR	1.36 (1.25–1.49)	1.14 (1.02–1.28)	2.04 (1.53–2.71)
	Previous CVD	1.48 (1.42–1.54)	1.12 (1.06–1.19)	3.35 (3.01–3.74)
Men	Low CVR	Reference	Reference	Reference
	Low-intermediate CVR	1.09 (1.03–1.16)	1.10 (1.02–1.18)	1.25 (1.03–1.51)
	High-intermediate CVR	1.15 (1.09–1.21)	1.13 (1.05–1.22)	1.45 (1.21–1.72)
	High CVR	1.28 (1.21–1.34)	1.21 (1.13–1.30)	1.86 (1.59–2.19)
	Previous CVD	1.60 (1.53–1.68)	1.25 (1.17–1.34)	3.87 (3.37–4.45)

*CVD* indicates cardiovascular disease, *CVR* cardiovascular risk

**Fig. 1:**
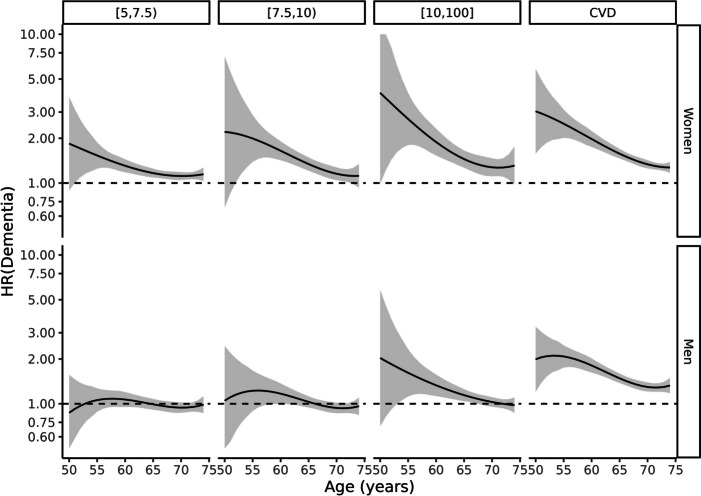
Cox model of all-cause dementia according to exposure groups by sex and age. The reference is a person with the same sex and age in the lowest risk group (Framingham-REGICOR risk < 5%)

**Fig. 2:**
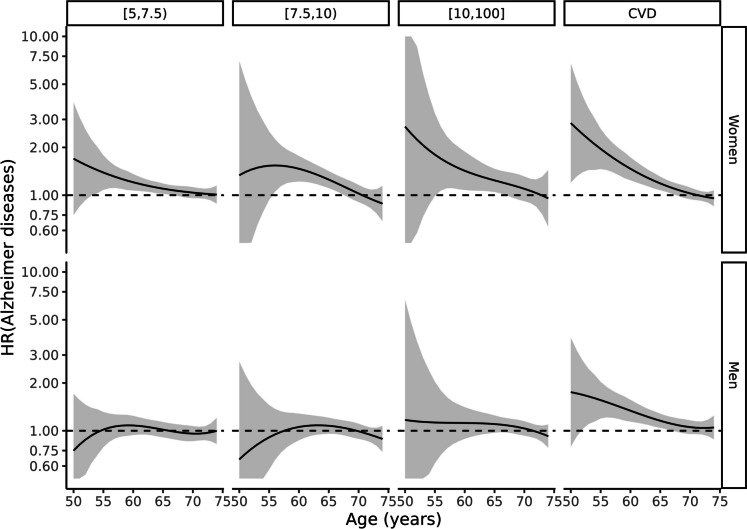
Cox model of Alzheimer’s disease according to exposure groups by sex and age. The reference is a person with the same sex and age in the lowest risk group (Framingham-REGICOR risk < 5%)

**Fig. 3:**
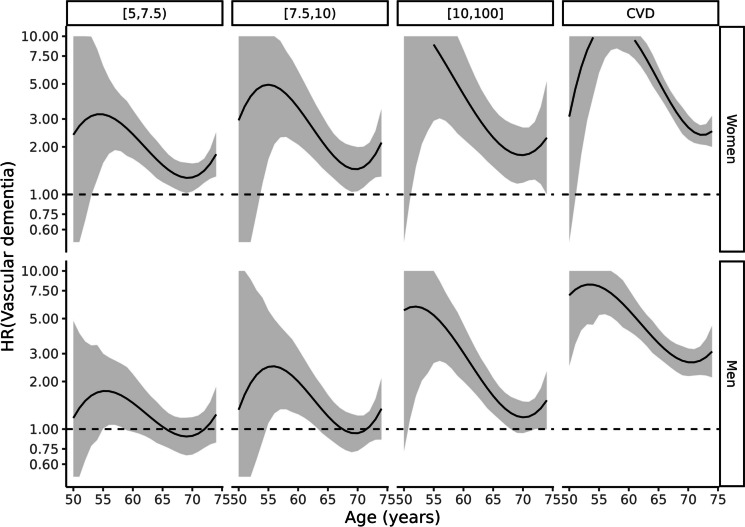
Cox model of vascular dementia according to exposure groups by sex and age. The reference is a person with the same sex and age in the lowest risk group (Framingham-REGICOR risk < 5%)

Spline models showed that the association between cardiovascular risk and dementia differed by age and sex (Figs. 1, 2, and 3); the *p*-value for the interaction was < 0.001 in all outcomes. In midlife, significant associations were stronger than in later life; and were more noticeable in women than in men (Figs. 1, 2, and 3). In women, cardiovascular risk is associated with dementia in all the exposure groups and dementia outcomes. But men only showed significant associations in the highest cardiovascular risk groups and regarding all-cause and vascular dementia (Figs. 1, 2, and 3). The significant HRs were clearly higher in women; for example, in people aged 53 years with high cardiovascular risk, the HR of all-cause dementia was 3.16 in women, and 1.79 in men. Moreover, women with a high Framingham-REGICOR score presented a similar risk of developing dementia — of any type — to women who had previous vascular disease (Figs. 1, 2, and 3). Finally, the age at which the HR remained significant was older in women than in men (Figs. 1, 2, and 3); for example, in the group with high cardiovascular risk, the HR for all-cause dementia were significant in women aged between 51 and 74 years, and in men aged between 53 and 68 years. The sensitivity analyses presented similar results (Figs. S2–S4, Table S5).

## Discussion

This mega-cohort study of about 1 million people and > 50,000 dementia cases based on routinely collected data from the primary care services provides a novel contribution: it shows a dose–response association between the Framingham-REGICOR score and the incidence of all-cause dementia, Alzheimer’s disease, and vascular dementia. This dose–response association was stronger in midlife and attenuated in later life, and more pronounced in women than in men. Women with a high Framingham-REGICOR score presented a similar risk of developing dementia — of any type — to women who had previous vascular disease; and at age 50–55, they showed three times higher risk of developing dementia than the lowest exposure group.

### Comparison with existing literature

We found that the highest risk of developing dementia later in life was at 50–55 years of age, and it decreased at older ages, in line with previous studies [6–8]. In fact, the pathological changes that lead to dementia onset might start decades before the clinical manifestations of cognitive impairment and dementia [28]. Regarding the potential role of cardiovascular risk factors on dementia, poor cardiovascular health at age 50 has been related with increased risk of dementia and brain volume 20 years later [5]. We observed an attenuated association between Framingham-REGICOR at older ages and later dementia onset, which could be consistent with reverse causation. Dementia symptoms are preceded by a preclinical phase (of about 20–30 years) during which levels of cardiovascular risk factors could decrease, resulting in an attenuation or even a reverse association between Framingham-REGICOR and dementia [29–31]. In this scenario, lower levels of cardiovascular risk factors at older ages would be a consequence of preclinical disease rather than a cause of dementia [5, 29].

Our results emphasize a sex-dependent association between the Framingham-REGICOR score and dementia onset. We observed that significant hazard ratios were higher and occurred until older ages in women, in all exposure groups. These results align with previous descriptions of a stronger association between cardiovascular risk scores and cognitive function [32, 33] or dementia [10] in women. However, two analyses found a similar association in both sexes [4, 5]. Their limited sample size could have hindered the detection of a significant association when assessing the effect of sex [4]. The observed sex differences in our study could be explained by the following reasons. First, we observed poorer cardiovascular health in women; within a given exposure group, women tended to have worse levels of total cholesterol, HDL-C, and a higher prevalence of hypertension, DM2, and obesity. These findings agree with previous studies that reported worse control of several cardiovascular risk factors in women [34]. In contrast, we observed that Framingham-REGICOR score increased with age in men, but not in women. Thus, aging might be a strong confounder of the association between Framingham-REGICOR and dementia in men. Second, the role of the individual cardiovascular risk factors on the dementia risk may differ by sex. Several studies reported a higher susceptibility in women to the effects of midlife elevated blood pressure [35, 36] or DM2 [6, 37, 38], which increased the risk of dementia. In our study, hypertension and DM2 were highly prevalent among women in most of the exposure groups; for example, in the high-risk group, the prevalence of DM2 reached 90.8% in women and 46.1% in men. In Catalonia, women with diabetes have shown worse target control than men [39, 40]. Moreover, the strongest impact of DM2 in women occurred in midlife and persisted after age 80 [6]. In our sample, women with intermediate or high cardiovascular risk were 63 years old on average; thus, in most of them, DM2 might still have a long time to contribute to dementia onset. Third, differences in genetics, hormones, structural brain development, or functional connectivity between men and women could contribute to the sex-related association between cardiovascular health and dementia we observed [37, 41]. Finally, certain gender roles might also contribute to the increased risk in women. Older women may not have had as many opportunities to receive a higher level of education or to do physical exercise as men, or they deliver caregiving tasks more frequently than men — these three aspects have been described as risk factors for both dementia [41] and cardiovascular disease [42].

Regarding dementia subtypes, we observed that cardiovascular risk was associated with vascular dementia and Alzheimer’s disease, in line with previous studies [4, 43–45]. The associations were stronger with vascular dementia; these are expected results because the main risk factors for vascular dementia are included in the REGICOR-Framingham score. Regarding Alzheimer’s disease, the vascular component is less evident; genetics plays an important role in the disease risk profile and may interact with the association between cardiovascular risk and dementia [4].

### Implications for practice and research

Overall, our findings suggest that general practitioners could use sex- and age-specific cardiovascular risk scores to convey the risk of developing dementia and recommend healthier lifestyles that could harness it. Our results indicate that middle-aged adults may be the target population: middle-aged men with Framingham-REGICOR scores ≥ 10, middle-aged women with Framingham-REGICOR scores ≥ 5, and patients with vascular disease. From a public health perspective, minor improvements in the cardiovascular risk score among women at low or intermediate risk could represent a big impact on preventing dementia at a population level. The provided sex-specific recommendations may help prevent gender biases when diagnosing or treating cardiovascular risk factors [46]. To researchers, the use of sex-specific cardiovascular risk scores may help integrate a necessary sex and gender perspective in future studies on the relationship between cardiovascular health and dementia.

### Strengths and limitations

The main strength of this study was the high external validity grounded on a large sample size and 15 years of follow-up, which is longer than that in previous studies [10, 43, 45]. Moreover, the analysis of electronic health records allows the study of real-life clinical conditions and precludes a selection bias due to low response rates or losses during follow-up, which could be common among people with dementia. The internal validity in this study was ensured through the use of validated records of cardiovascular risk factors [15], overall dementia [12], and Alzheimer’s disease [16].

We also acknowledge several limitations. First, there could be some residual confounding due to unobserved variables that were not available in SIDIAP, such as educational level [47] or physical activity [43]. Second, under-registration of certain health records might promote selection bias, but we mitigated this limitation by imputing the missing data. Finally, the vascular dementia records were not validated, but the observed dose–response association between the REGICOR-Framingham score and vascular dementia may indicate a reasonable accuracy of the diagnoses.

## Conclusions

Our findings highlight that the Framingham-REGICOR scores are associated with the incidence of overall dementia, Alzheimer’s disease, and vascular dementia, in a dose-dependent way. The presence of a dose–response association between cardiovascular risk score and dementia onset is compatible with causality. This association is stronger in midlife, attenuated in older ages, and more pronounced in women. Women with high estimates of the Framingham-REGICOR score presented a risk of developing dementia similar to women with a history of cardiovascular disease. We would recommend that general practitioners use sex-and-age-specific scores to assess cardiovascular risk to prevent dementia, and public health managers incorporate gender perspective into the policies to improve cardiovascular health in midlife and prevent dementia later in life.

### Supplementary Information

**Additional file 1:** **Box S1.** Definition of exclusion criteria. **Box S2.** Definition of exposure group with previous cardiovascular disease. **Box S3.** Definition of cases of overall dementia, Alzheimer’s disease, and vascular dementia. **Box S4.** Definition of baseline covariates. **Box S5.** Validation of the imputation process. **Table S1.** Description of missing,complete case and imputed datasets. **Table S2.** Description of the baseline characteristics of the study population by sex. **Table S3.** Crude incidences (95% CI) of Alzheimer’s disease by sex, age and exposure groups. **Table S4.** Crude incidences (95% CI) of vascular dementia by sex, age and exposure groups. **Table S5.** Subdistribution hazard ratios (95% CI) of the exposure groups obtained in the Fine-Gray models. **Figure S1.** Flowchart of the study population. **Figure S2.** Unadjusted Cox model b-splines of hazard ratios of dementia types (panel A: all-cause dementia; B: Alzheimer’s disease; C: vascular dementia) according to exposure groups by sex and age. **Figure S3. **Complete case analysis to replicate the Cox model b-splines of hazard ratios of dementia types (panel A: all-cause dementia; B: Alzheimer’s disease; C: vascular dementia) according to exposure groups by sex and age. **Figure S4. **Cox model b-splines of hazard ratios of dementia types (panel A: all-cause dementia; B: Alzheimer’s disease; C: vascular dementia) according to exposure groups by sex and age. Censuring was applied at the first cardiovascular event or dementia onset during follow-up.

## Data Availability

The datasets generated and analyzed during the current study are not publicly available due to legal reasons related to data privacy protection but are available from the corresponding author on reasonable request.

## Abbreviations

CVR: Cardiovascular risk
SIDIAP: System for the Development of Research in Primary Care
ICD-10-CM: International Classification of Diseases, tenth revision, clinical modification
ATC: Anatomical Therapeutic Chemical Classification code
DM2: Type-2 diabetes mellitus
