# Supplementary material for: Sex matters in the association between cardiovascular health and incident dementia: evidence from real world data
Source: Alzheimers Res Ther. 2024 Mar 14;16:58. doi: 10.1186/s13195-024-01406-x (PMC10938682; doi:10.1186/s13195-024-01406-x)

**ADDITIONAL MATERIAL**

**Box S1. Definition of exclusion criteria**

Excluded patients were those treated with antidementia drugs (Anatomical Therapeutic Chemical Classification, ATC: N06DA i N06DX01) or diagnosed for dementia, cognitive impairment, or mental illness (ICD-10-CM codes: F11, F12, F13, F14, F15, F16, F17, F18, F19, F05, F53.1, F59, A52.1, A52.3, E52, E53.9, E83.0, F04, F06, F09, G35, F07, F80, F81, F82, F84, F88, F89, F70, f71, F72, F73, F78, F79, R41.8) before baseline; and users who did not attend the primary care services up to one year before baseline.

**Box S2. Definition of exposure group with previous vascular disease**

Patients who had one of the following conditions (ICD-10-CM codes) recorded in their electronic health records prior to or at baseline were considered as having previous cardiovascular disease: angina pectoris (I20), ischemic heart diseases (I20-I25), stroke (I60-I64), peripheral artery disease (I70, I70.2, I70.8, I70.9, I73, I73.8, I73.9, I74.3), or transient cerebral ischemic (G45-G46).

**Box S3. Definition of cases**

The definitions of the considered dementia types are described hereunder.

**Overall dementia**: patients diagnosed for dementia (ICD10: dementia in Alzheimer’s disease (F00), vascular dementia (F01), dementia in other diseases classified elsewhere (F02.8), unspecified dementia (F03), Alzheimer’s disease (G30), Lewy body disease (G31.8), or frontotemporal dementia (G31.0)), or treated with anti-dementia drugs (ATC: anticholinesterases (N06DA) or memantine (N06DX01)). This definition has been validated (positive predictive value 91.0% (95%CI: 87.5%-94.5%)) (Ponjoan et al. 2019).

**Alzheimer’s disease:** patients who have a code for Alzheimer’s disease diagnosis (ICD10: F00 or G30) or for prescription or billing of anti-dementia drugs (ATC: N06DA, N06DX01). Treated patients were included if they had no code for dementia diagnosis or had a code for unspecified dementia (F03), and were excluded if they had a code for: a specific subtype of dementia such as Lewy bodies dementia, vascular or frontotemporal dementia (ICD10: F01, F02); Parkinson (ICD10: G20-G22); anti-Parkinson drugs (ATC: N04); or cerebrovascular disease (ICD10:I60- I69, G45, G46) within two years prior to an Alzheimer’s disease diagnosis.

**Vascular dementia:** patients who have a code for vascular dementia diagnosis (ICD10: F01).

**Box S4. Definition of baseline covariates**

Further details on the definition of covariates are described hereunder.

- Rurality (rural/urban). We considered as rural those municipalities with <10,000 inhabitants and a population density <150/km^2^. This criterion was defined by the Catalan Government:

Generalitat de Catalunya. Departament d’Agricultura, Ramaderia, Pesca, Alimentació i Medi Natural. Programa de desarrollo rural de Catalunya. Período de programación 2007-2013. Decisión C. Published online 2008.

- Comorbidities were defined using the ICD10 diagnostic codes: DM2 (E10 and E11), hyperthyroidism (E05), alcohol-related disorders (F10), Parkinson’s disease (G21), depression (F30-F34 and F39).
- Smoking: currently smoker or former smoker for less than 1 year; former smoker for more than 1 year or never smoked.
- Obesity: defined as having a diagnostic code (ICD10: E66) or a body mass index higher than 30 kg/m^2^.

**Box S5. Validation of the imputation process**

In the pre-multiple imputation stage, we checked for normality, extreme values, and outliers of all the continuous variables, and correlations and collinearity of those with missing data and those that may be used in the multiple imputation. Variables related to the data being missing and/or to the value of missing data were identified. Based on these results, we assumed that the missingness mechanism was at random for each of the variables to be imputed and was related to different subsets of the variables included in the imputation models. That is, it was possible to estimate their missing values as conditioned by the values of other observed variables. The following variables were included in the imputation models: age, number of visits in the previous year, smoking, diabetes, hypertension, dyslipidemia, atrial fibrillation, arthritis, asthma, chronic obstructive pulmonary disease, alcoholism, hyper- and hypothyroidism, neoplasms, obesity, anemia, depression, Parkinson disease, 'Endocrine, nutritional and metabolic diseases', 'Need for assistance at home and no household member able to render care', 'Problems related to living in a residential institution', antihypertensive agents, diuretics, beta-blocking agents, calcium-channel blockers, agents acting on the renin angiotensin aldosterone system, hypoglycemic agents, statins, antiplatelet agents, analgesics, anti-inflammatory and antirheumatic agents, psycholeptics, and the natural logarithm (ln) of the systolic blood pressure, diastolic blood pressure, total cholesterol, high density lipoprotein cholesterol, and triglycerides. We also included incident dementia disease and time to incident dementia disease or time to censoring.

We used the natural logarithmic transformation of the indicated continuous variables included in the imputation models to improve normality of distribution and to avoid the unlikely possibility of imputing any negative numbers. After imputation, variables were transformed back to the original scale.

Our study analyzed general population data from electronic health records, where the missing data are missing at random (MAR). The imputation process carried out in this study had to include multiple interaction terms between categorical variables, to mitigate a potential bias (Tilling *et al.* 2016; J Clin Epidemiol. 2016 Dec;80:107-115). As shown by Tilling et al. 2016, imputation models that integrate interactions with categorical variables can be conceptualized as stratified models, ensuring an unbiased process. The imputation process was therefore stratified by sex, previous hypertension (HTA), diabetes (DM), or cardiovascular disease (CVD). The 10 subpopulations are: women with previous DM and HTA and without CVD; women with previous DM and without CVD and HTA; women with previous HTA and without DM and CVD; women without previous DM, CVD, and HTA; men with previous CVD; men with previous DM and HTA and without CVD; men with previous DM and without CVD and HTA; men with previous HTA and without DM and CVD; men without previous DM, CVD, and HTA.

**Table S1.** Description of missing values, complete cases, and imputed datasets.

| **Variable** | **Missing Counts**  N(%) | **Complete cases**  N(%), mean[sd] | **Incomplete cases**  N(%), mean[sd] | **p-value**^†^ | **Imputed**  **dataset**  N(%), mean[sd] |
| --- | --- | --- | --- | --- | --- |
| N | 922973 | 302161 | 620812 |  | 922973 |
| Years of follow-up | 0 (0.0%) | 12.4 [3.3] | 12.5 [3.4] | <0.001 | 12.5 [3.3] |
| Framingham-REGICOR | 473515 (51.3%) | 4.8 [3.4] | 4.3 [3.0] | <0.001 | 4.2 [3.0] |
| Exposure groups* | 473515 (51.3%) |  |  | <0.001 |  |
| Low CVR | - | 183355 (60.7 %) | 83182 (56.5%) |  | 641582 (69.5%) |
| Low-intermediate CVR | - | 47515 (15.7 %) | 17000 (11.5%) |  | 124566 (13.5%) |
| High-intermediate CVR | - | 22367 (7.4%) | 7074 (4.8%) |  | 51998 (5.6%) |
| High CVR | - | 19404 (6.4%) | 5342 (3.6%) |  | 40608 (4.4%) |
| Previous Vascular Disease | - | 29520 (9.8%) | 34699 (23.6%) |  | 64219 (7.0%) |
| Age (years) | 0 (0.0%) | 63.6 [6.8] | 60.9 [7.1] | <0.001 | 61.7 [7.1] |
| Total cholesterol^#^ (mg/dl) | 290767 (31.5%) | 209.8 [37.8] | 212.8 [37.1] | <0.001 | 211.1 [36.9] |
| HDL^#^ (mg/dl) | 413510 (44.8%) | 56.5 [14.6] | 58.5 [15.2] | <0.001 | 57.6 [14.9] |
| Triglycerides^#^ (mg/dl) | 356073 (38.6%) | 125.1 [68.0] | 123.6 [86.7] | <0.001 | 120.3 [74.0] |
| Systolic BP^#^ (mmHg) | 278627 (30.2%) | 133.8 [15.4] | 132.1 [15.6] | <0.001 | 131.9 [15.3] |
| Diastolic BP^#^ (mmHg) | 278617 (30.2%) | 77.9  [9.1] | 78.0  [9.3] | <0.001 | 77.8  [9.2] |
| Weight^#^ (kg) | 451128 (48.9%) | 75.9  [13.9] | 75.2  [14.2] | <0.001 | 74.2  [13.7] |
| Height^#^ (cm) | 113187 (12.3%) | 159.8  [9.1] | 161.5  [9.3] | <0.001 | 160.9  [9.3] |
| Hypertension | 0 (0.0%) | 179554 (59.4%) | 178463  (28.7%) | <0.001 | 358017  (38.8%) |
| Smoking | 0 (0.0%) | 35102  (11.6%) | 91496  (14.7%) | <0.001 | 126598  (13.7%) |
| DM2 | 0 (0.0%) | 80861  (26.8%) | 50291  (8.1%) | <0.001 | 131152  (14.2%) |
| Obesity | 0 (0.0%) | 79982  (26.5%) | 77216  (12.4%) | <0.001 | 157198  (17.0%) |
| Depression | 0 (0.0%) | 19546  (6.5%) | 36277  (5.8%) | <0.001 | 55823  (6.0%) |
| Hyperthyroidism | 0 (0.0%) | 2687  (0.9%) | 4439  (0.7%) | <0.001 | 7126  (0.8%) |
| Alcohol disorders | 0 (0.0%) | 6791  (2.2%) | 10671  (1.7%) | <0.001 | 17462  (1.9%) |
| Rurality | 0 (0.0%) | 47790 (15.8%) | 105227 (16.9%) | <0.001 | 153017  (16.6%) |
| Incident CVD | 0 (0.0%) | 61033 (20.2%) | 88866  (14.3%) | <0.001 | 149899  (16.2%) |

|  |  |  |  |
| --- | --- | --- | --- |
|  |  |  |  |
|  |  |  |  |
|  |  |  |  |
|  |  |  |  |
|  |  |  |  |
|  |  |  |  |
|  |  |  |  |
|  |  |  |  |
|  |  |  |  |
|  |  |  |  |
|  |  |  |  |
|  |  |  |  |
|  |  |  |  |
|  |  |  |  |
|  |  |  |  |
|  |  |  |  |
|  |  |  |  |
|  |  |  |  |
|  |  |  |  |
|  |  |  |  |
|  |  |  |  |
|  |  |  |  |
|  |  |  |  |
|  |  |  |  |
|  |  |  |  |
|  |  |  |  |

*Exposure groups based on cardiovascular risk (CVR) at baseline: low (Framingham-REGICOR<5%), low- intermediate (5%≤Framingham -REGICOR <7.5%), high-intermediate (7.5%≤ Framingham-REGICOR<10%), high (Framingham-REGICOR ≥10%), and patients with history of vascular disease previous to the baseline.

^#^Imputed variables.

**^†^** P-value to compare the complete case and the incomplete case datasets.

**Table S2**. Description of the baseline characteristics of the study population by sex.

|  |  | Total  n(%); mean[sd] | Women  n(%); mean[sd] | Men  n(%); mean[sd] | p_valor |
| --- | --- | --- | --- | --- | --- |
| Years of follow-up |  | 12.5 ([3.3] | 12.7 [3.1] | 12.1 [3.6] | <0.001 |
| Framingham-REGICOR |  | 4.2 [3.0] | 3.2 [2.0] | 5.7 [3.7] | <0.001 |
| Exposure groups* |  |  |  |  |  |
| Low CVR |  | 641582 (69.5) | 462824 (83.7) | 178757 (48.3) | <0.001 |
| Low-intermediate CVR |  | 124566 (13.5) | 47482 (8.6) | 77084 (20.8) | <0.001 |
| High-intermediate CVR |  | 51998 (5.6) | 13462 (2.4) | 38536 (10.4) | <0.001 |
| High CVR |  | 40608 (4.4) | 6863 (1.2) | 33745 (9.1) | <0.001 |
| Previous Vascular Disease |  | 64219 (7.0) | 22415 (4.1) | 41804 (11.3) | <0.001 |
| Age (years) |  | 61.7 [7.1] | 61.8 [7.1] | 61.7 [7.1] | 0.223 |
| Total cholesterol (mg/dl) |  | 211.1 [36.9] | 216.1 [35.6] | 203.7 [37.6] | <0.001 |
| HDL (mg/dl) |  | 57.6 [14.9] | 61.8 [14.8] | 51.2 [12.7] | <0.001 |
| Systolic BP (mmHg) |  | 131.9 [15.3] | 130.8 [15.3] | 133.6 [15.2] | <0.001 |
| Diastolic BP (mmHg) |  | 77.8 [9.2] | 77.3 [9.1] | 78.6 [9.3] | <0.001 |
| BMI (kg/m²) |  | 28.8 [5.3] | 29.0 [5.7] | 28.5 [4.8] | <0.001 |
| Hypertension |  | 358017 (38.8) | 212629 (38.4) | 145388 (39.3) | <0.001 |
| Smoking |  | 126598 (13.7) | 41397 (7.5) | 85201 (23.0) | <0.001 |
| DM2 |  | 131152 (14.2) | 66314 (12.0) | 64838 (17.5) | <0.001 |
| Obesity |  | 157198 (17.0) | 111129 (20.1) | 46069 (12.5) | <0.001 |
| Depression |  | 55823 (6.0) | 43883 (7.9) | 11940 (3.2) | <0.001 |
| Hyperthyroidism |  | 7126 (0.8) | 5972 (1.1) | 1154 (0.3) | <0.001 |
| Alcohol disorders |  | 17462 (1.9) | 2907 (0.5) | 14555 (3.9) | <0.001 |
| Rurality |  | 153017 (16.6) | 88114 (15.9) | 64903 (17.5) | <0.001 |
| Dyslipidemia medication ^#^ |  | 239814 (26.0) | 137992 (25.0) | 101822 (27.5) | <0.001 |
| Hypertension medication^##^ |  | 362296 (39.3) | 210670 (38.1) | 151626 (41.0) | <0.001 |
| Incident CVD |  | 149899 (16.2) | 67120 (12.1) | 82779 (22.4) | <0.001 |

*Exposure groups based on cardiovascular risk (CVR) at baseline: low (Framingham-REGICOR<5%), low- intermediate (5%≤Framingham-REGICOR<7.5%), high-intermediate (7.5%≤ Framingham-REGICOR<10%), high (Framingham-REGICOR ≥10%), and patients with history of vascular disease previous at baseline.

^#^Dyslipidemia medication: prescription of lipid modifying agents.

^##^Hypertensive medication: prescription of antihypertensives, Diuretics, Beta blocking agents, Calcium channel blockers or Agents acting on the renin–angiotensin system.

**Table S3.** Crude incidences (95% CI) of Alzheimer’s disease by sex, age, and exposure groups.

| **Sex** | **Age** | **Whole population** | **Exposure groups^*^** (risk at baseline) n(%); mean[sd] | | | | |
| --- | --- | --- | --- | --- | --- | --- | --- |
|  |  |  | **Low**  **CVR** | **Low-**  **Intermediate CVR** | **high- Intermediate CVR** | **High CVR** | **Previous**  **vascular disease** |
| Women | [50,55) | 0.27 (0.24 - 0.29) | 0.25 (0.23 - 0.28) | 0.39 (0.25 - 0.60) | 0.63 (0.30 - 1.32) | 0.97 (0.36 - 2.58) | 0.51 (0.26 - 0.97) |
|  | [55,60) | 0.74 (0.70 - 0.79) | 0.70 (0.66 - 0.75) | 0.94 (0.78 - 1.13) | 1.08 (0.77 - 1.52) | 1.09 (0.67 - 1.78) | 1.36 (1.01 - 1.85) |
|  | [60,65) | 2.24 (2.16 - 2.31) | 2.14 (2.06 - 2.22) | 2.50 (2.28 - 2.74) | 2.86 (2.43 - 3.37) | 2.92 (2.35 - 3.64) | 2.78 (2.36 - 3.26) |
|  | [65,70) | 4.96 (4.84 - 5.08) | 4.90 (4.76 - 5.04) | 5.08 (4.72 - 5.45) | 5.59 (4.94 - 6.32) | 4.82 (4.01 - 5.80) | 5.24 (4.71 - 5.82) |
|  | [70,75) | 9.25 (9.08 - 9.42) | 9.33 (9.14 - 9.52) | 8.72 (8.17 - 9.31) | 8.75 (7.72 - 9.92) | 8.75 (7.22 - 10.61) | 9.16 (8.56 - 9.79) |
|  | All | 3.20 (3.16 - 3.25) | 3.02 (2.98 - 3.07) | 3.75 (3.58 - 3.93) | 4.00 (3.68 - 4.34) | 3.97 (3.54 - 4.44) | 5.34 (5.07 - 5.63) |
| Men | [50,55) | 0.21 (0.19 - 0.25) | 0.21 (0.18 - 0.24) | 0.24 (0.17 - 0.35) | 0.13 (0.05 - 0.36) | 0.18 (0.04 - 0.71) | 0.32 (0.18 - 0.54) |
|  | [55,60) | 0.54 (0.49 - 0.58) | 0.49 (0.44 - 0.55) | 0.55 (0.45 - 0.67) | 0.52 (0.36 - 0.73) | 0.65 (0.43 - 0.99) | 0.87 (0.67 - 1.12) |
|  | [60,65) | 1.55 (1.48 - 1.63) | 1.42 (1.32 - 1.53) | 1.70 (1.54 - 1.87) | 1.61 (1.39 - 1.87) | 1.60 (1.33 - 1.91) | 1.73 (1.50 - 1.99) |
|  | [65,70) | 3.31 (3.19 - 3.44) | 3.15 (2.94 - 3.37) | 3.17 (2.94 - 3.41) | 3.46 (3.15 - 3.81) | 3.66 (3.32 - 4.04) | 3.49 (3.16 - 3.85) |
|  | [70,75) | 6.31 (6.13 - 6.49) | 6.41 (5.99 - 6.85) | 6.17 (5.81 - 6.54) | 6.12 (5.73 - 6.55) | 6.27 (5.90 - 6.67) | 6.67 (6.24 - 7.13) |
|  | All | 2.16 (2.11 - 2.20) | 1.25 (1.20 - 1.31) | 2.48 (2.36 - 2.59) | 3.25 (3.07 - 3.44) | 4.01 (3.80 - 4.23) | 3.28 (3.12 - 3.45) |

*Exposure groups based on cardiovascular risk (CVR) at baseline: low (Framingham-REGICOR<5%), low- intermediate (5%≤Framingham-REGICOR<7.5%), high-intermediate (7.5%≤ Framingham-REGICOR<10%), high (Framingham-REGICOR ≥10%), and patients with history of vascular disease previous at baseline.

**Table S4.** Crude incidences (95% CI) of vascular dementia by sex, age, and exposure groups.

| **Sex** | **Age** | **Whole population** | **Exposure groups^*^** (risk at baseline) n(%); mean[sd] | | | | |
| --- | --- | --- | --- | --- | --- | --- | --- |
|  |  |  | **Low**  **CVR** | **Low-**  **Intermediate CVR** | **high- Intermediate CVR** | **High CVR** | **Previous**  **vascular disease** |
| Women | [50,55) | 0.02 (0.01 - 0.03) | 0.02 (0.01 - 0.02) | 0.02 (0.00 - 0.14) | 0.27 (0.09 - 0.83) | 0.24 (0.03 - 1.71) | 0.11 (0.03 - 0.45) |
|  | [55,60) | 0.06 (0.05 - 0.07) | 0.04 (0.03 - 0.05) | 0.14 (0.08 - 0.22) | 0.06 (0.02 - 0.25) | 0.34 (0.14 - 0.81) | 0.39 (0.22 - 0.68) |
|  | [60,65) | 0.19 (0.17 - 0.21) | 0.13 (0.12 - 0.15) | 0.24 (0.18 - 0.32) | 0.34 (0.21 - 0.54) | 0.39 (0.22 - 0.71) | 1.07 (0.82 - 1.39) |
|  | [65,70) | 0.49 (0.45 - 0.53) | 0.39 (0.35 - 0.43) | 0.62 (0.51 - 0.77) | 0.63 (0.44 - 0.90) | 0.71 (0.44 - 1.14) | 1.58 (1.31 - 1.92) |
|  | [70,75) | 1.18 (1.13 - 1.25) | 1.00 (0.95 - 1.07) | 1.43 (1.23 - 1.68) | 1.72 (1.30 - 2.27) | 2.21 (1.52 - 3.22) | 2.54 (2.23 - 2.87) |
|  | All | 0.36 (0.34 - 0.37) | 0.28 (0.26 - 0.29) | 0.49 (0.43 - 0.56) | 0.61 (0.50 - 0.75) | 0.68 (0.52 - 0.90) | 1.58 (1.44 - 1.74) |
| Men | [50,55) | 0.05 (0.03 - 0.06) | 0.04 (0.02 - 0.05) | 0.03 (0.01 - 0.09) | 0.10 (0.03 - 0.31) | 0.09 (0.01 - 0.63) | 0.22 (0.11 - 0.42) |
|  | [55,60) | 0.12 (0.10 - 0.14) | 0.06 (0.04 - 0.08) | 0.12 (0.08 - 0.18) | 0.10 (0.04 - 0.22) | 0.36 (0.20 - 0.63) | 0.54 (0.39 - 0.75) |
|  | [60,65) | 0.31 (0.27 - 0.34) | 0.18 (0.15 - 0.22) | 0.24 (0.19 - 0.31) | 0.41 (0.31 - 0.55) | 0.39 (0.27 - 0.56) | 0.86 (0.70 - 1.05) |
|  | [65,70) | 0.64 (0.59 - 0.70) | 0.49 (0.41 - 0.58) | 0.48 (0.40 - 0.58) | 0.44 (0.34 - 0.57) | 0.72 (0.58 - 0.90) | 1.46 (1.26 - 1.70) |
|  | [70,75) | 1.52 (1.43 - 1.61) | 0.97 (0.82 - 1.15) | 1.16 (1.01 - 1.32) | 1.21 (1.04 - 1.40) | 1.48 (1.30 - 1.68) | 3.00 (2.71 - 3.31) |
|  | All | 0.47 (0.46 - 0.50) | 0.19 (0.17 - 0.21) | 0.41 (0.36 - 0.47) | 0.61 (0.54 - 0.69) | 0.92 (0.82 - 1.03) | 1.51 (1.40 - 1.62) |

*Exposure groups based on cardiovascular risk (CVR) at baseline: low (Framingham-REGICOR<5%), low- intermediate (5%≤Framingham-REGICOR<7.5%), high-intermediate (7.5%≤ Framingham-REGICOR<10%), high (Framingham-REGICOR ≥10%), and patients with history of vascular disease previous at baseline.

**Table S5.** Subdistribution hazard ratios (95% CI) of the exposure groups obtained in the Fine-Gray models.

| Population | Exposure groups | All-cause dementia | Alzheimer’s disease | Vascular dementia |
| --- | --- | --- | --- | --- |
| Overall | Low CVR | Reference | Reference | Reference |
|  | Low-intermediate CVR | 1.09 (1.06 – 1.13) | 1.04 (1.01 - 1.09) | 1.32 (1.19 - 1.48) |
|  | High-intermediate CVR | 1.13 (1.09 – 1.17) | 1.06 (1.01 - 1.12) | 1.48 (1.31 - 1.67) |
|  | High CVR | 1.21 (1.16 – 1.26) | 1.09 (1.03 - 1.16) | 1.79 (1.57 - 2.04) |
|  | Previous CVD | 1.34 (1.30 – 1.38) | 1.02 (0.98 - 1.06) | 3.21 (2.95 - 3.48) |
| Women | Low CVR | Reference | Reference | Reference |
|  | Low-intermediate CVR | 1.10 (1.06 – 1.15) | 1.03 (0.97 - 1.08) | 1.41 (1.23 - 1.64) |
|  | High-intermediate CVR | 1.14 (1.07 – 1.22) | 1.03 (0.94 - 1.12) | 1.61 (1.29 - 2.00) |
|  | High CVR | 1.24 (1.14 – 1.35) | 1.04 (0.93 - 1.16) | 1.85 (1.39 - 2.46) |
|  | Previous CVD | 1.31 (1.26 – 1.37) | 1.00 (0.94 - 1.05) | 2.98 (2.67 - 3.33) |
| Men | Low CVR | Reference | Reference | Reference |
|  | Low-intermediate CVR | 1.09 (1.03 – 1.15) | 1.09 (1.02 - 1.17) | 1.24 (1.02 - 1.52) |
|  | High-intermediate CVR | 1.13 (1.07 – 1.19) | 1.11 (1.03 - 1.19) | 1.43 (1.20 - 1.71) |
|  | High CVR | 1.21 (1.15 – 1.27) | 1.14 (1.06 - 1.23) | 1.78 (1.51 - 2.10) |
|  | Previous CVD | 1.37 (1.31 – 1.44) | 1.07 (1.00 - 1.15) | 3.34 (2.89 - 3.86) |

CVD indicates cardiovascular disease; CVR, cardiovascular risk.

**Figure S1.** Flowchart of the study population.

Catalan population in 2009

n = 7.475.420

Catalan population aged 50-74 years in 2009

n = 1.861.326

SIDIAP population aged 50-74 years in 2009

n = 1.200.700

SIDIAP population aged 50-74 years in 2009 with a previous visit in primary care services 1 year before baseline

n = 926.628

SIDIAP population fulfilling inclusion criteria

n = 922.973

Excluded (n=274.072): without previous visit in primary care 1 year before baseline

Excluded (n=3.655): Prevalent dementia (n = 2.464)

Prevalent mental disorders (n = 1.191)

Exposure groups

(Cardiovascular risk at baseline)

CVD: patients with previous cardiovascular disease (N = 64.219)

High: Framingham-REGICOR≥10% (N = 40.608)

High-Intermediate: 7.5%≤Framingham-REGICOR<10% (N = 51.998)

Low-Intermediate: 5%≤ Framingham-REGICOR<7.5% (N = 124.566)

Low: Framingham-REGICOR < 5% (N = 641.582)

**Figure S2.** Unadjusted Cox model b-splines of hazard ratios of dementia types (panel A: all-cause dementia; B: Alzheimer’s disease; C: vascular dementia) according to exposure groups by sex and age.

**Panel A: All-cause dementia
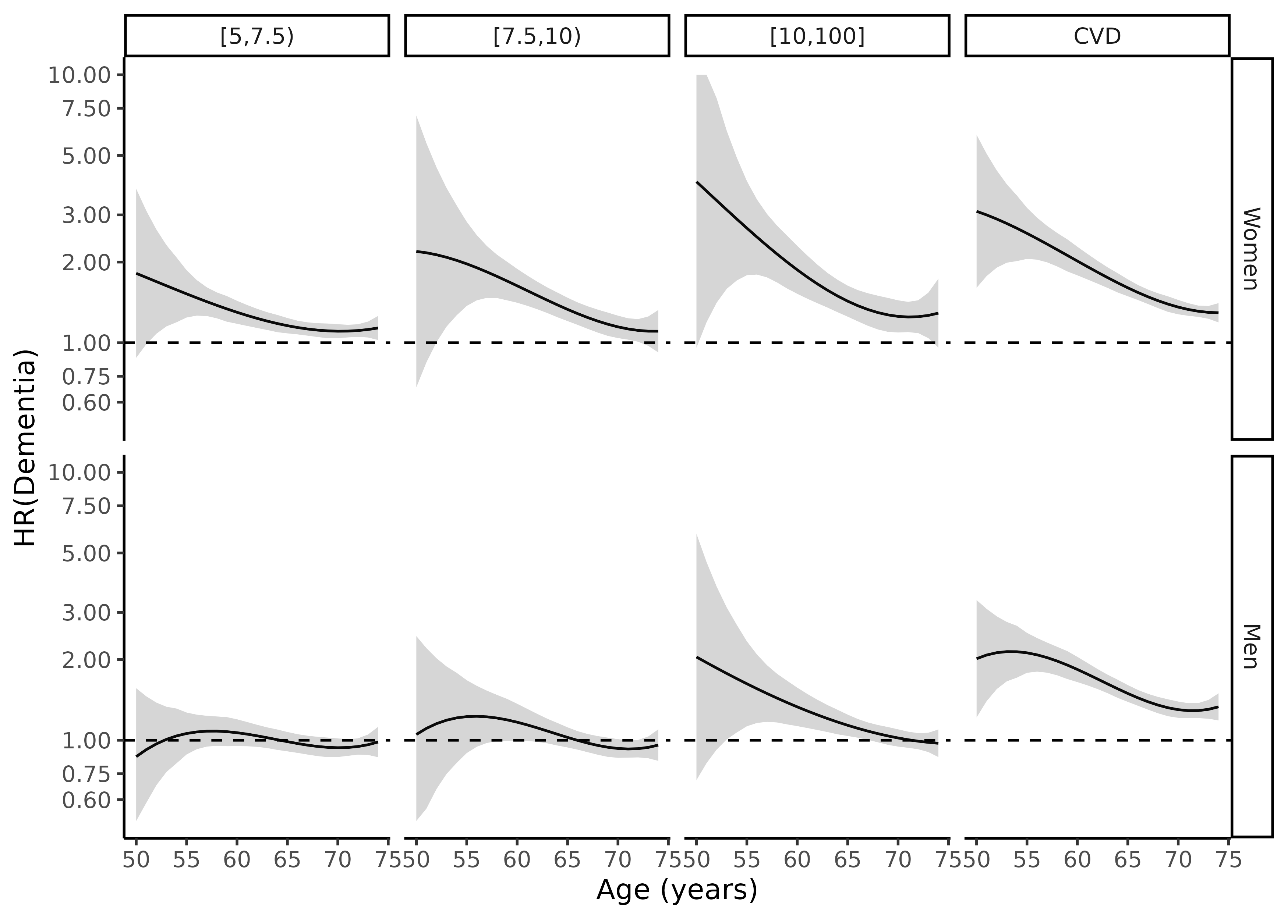
**

**Panel B: Alzheimer’s disease
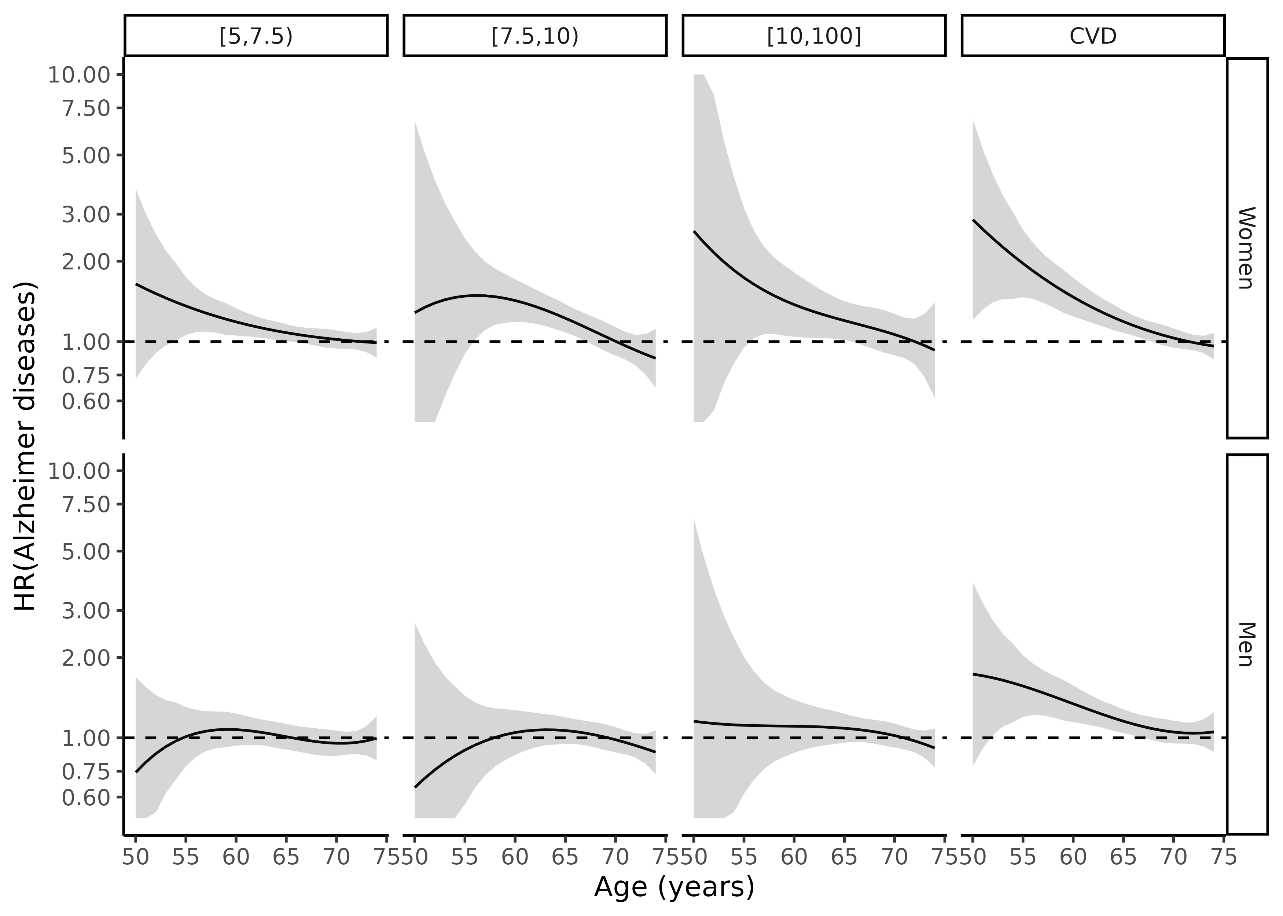
**

**Panel C: Vascular dementia**

**
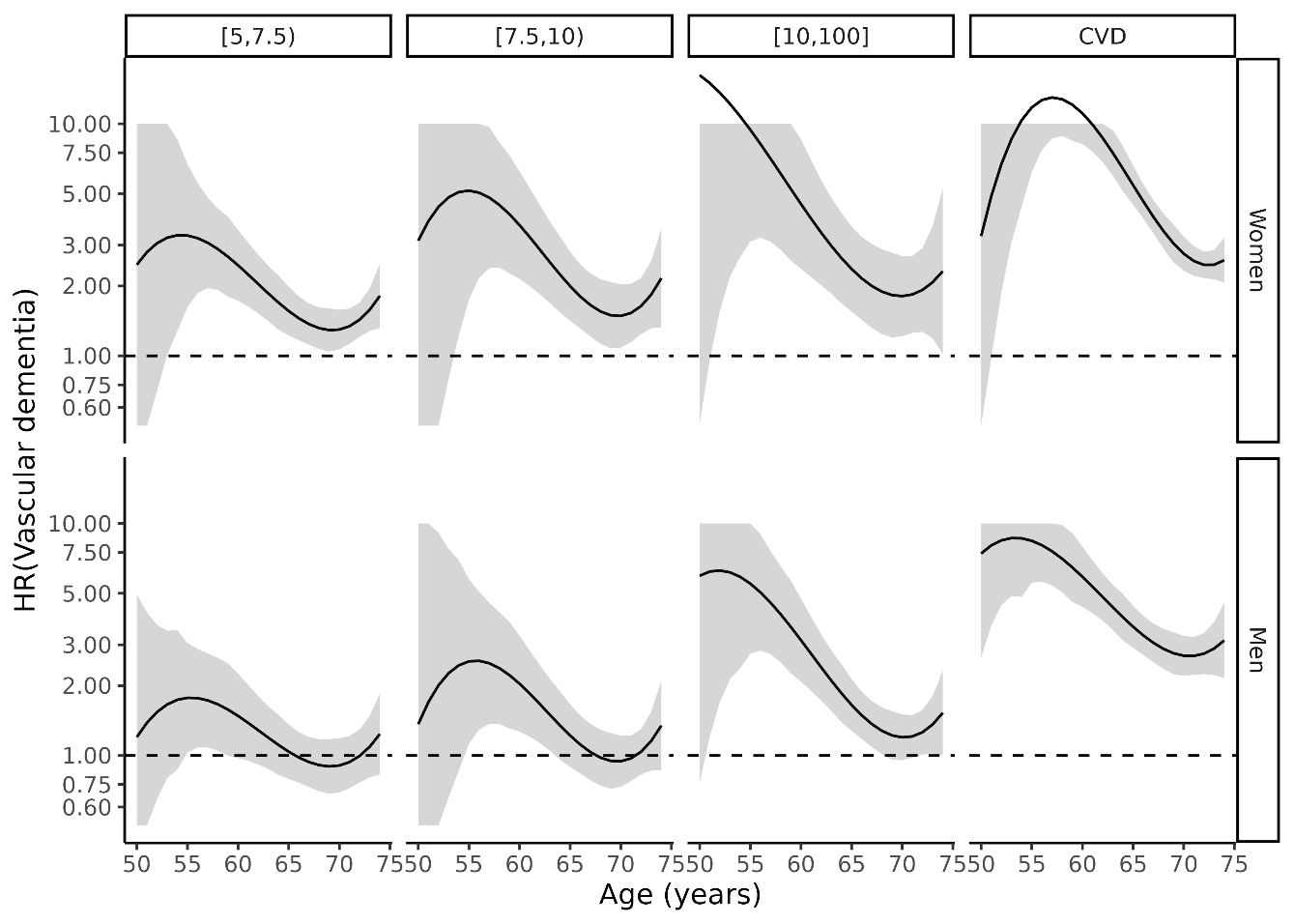
**

**Figure S3.** Complete case analysis to replicate the Cox model b-splines of hazard ratios of dementia types (panel A: all-cause dementia; B: Alzheimer’s disease; C: vascular dementia) according to exposure groups by sex and age.

**Panel A: all-cause dementia**

**
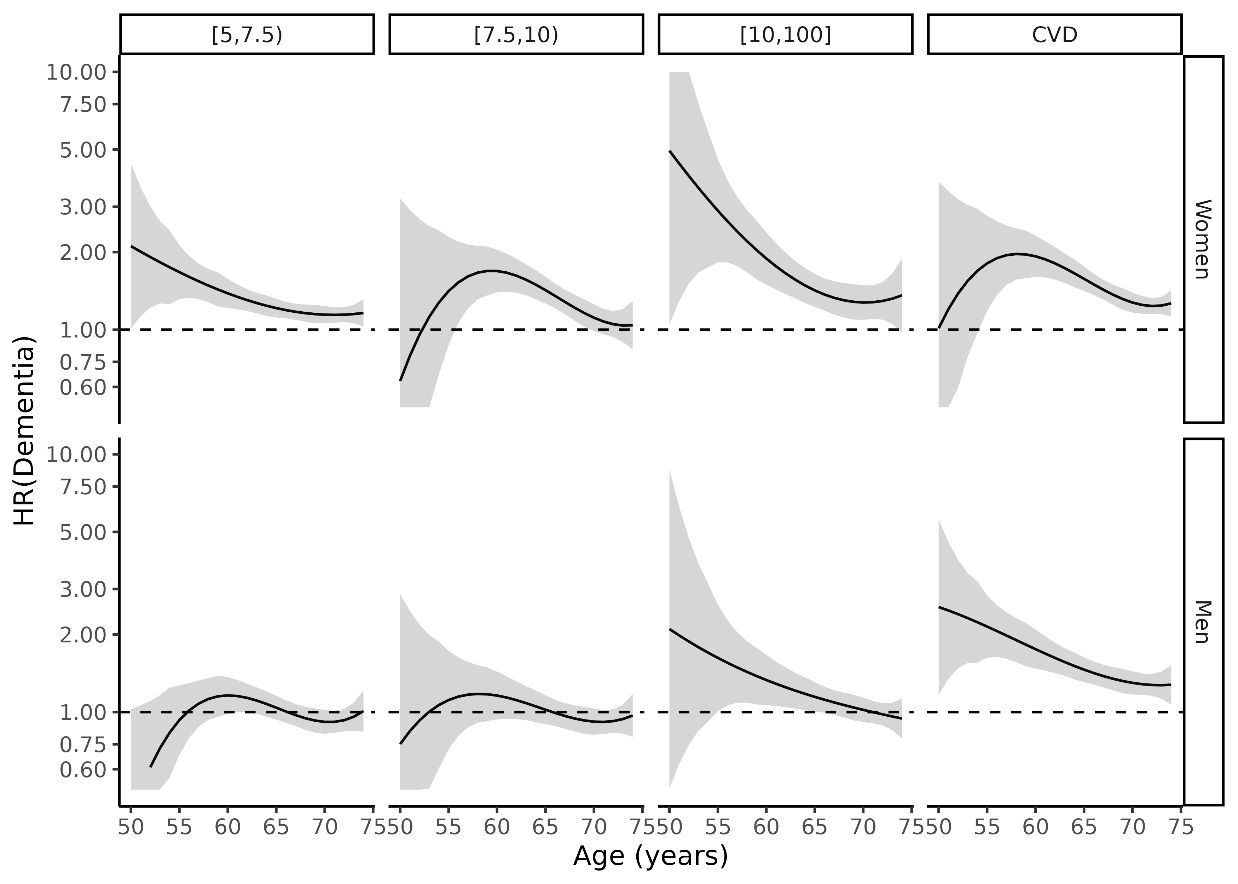
**

**Panel B: Alzheimer’s disease**

**
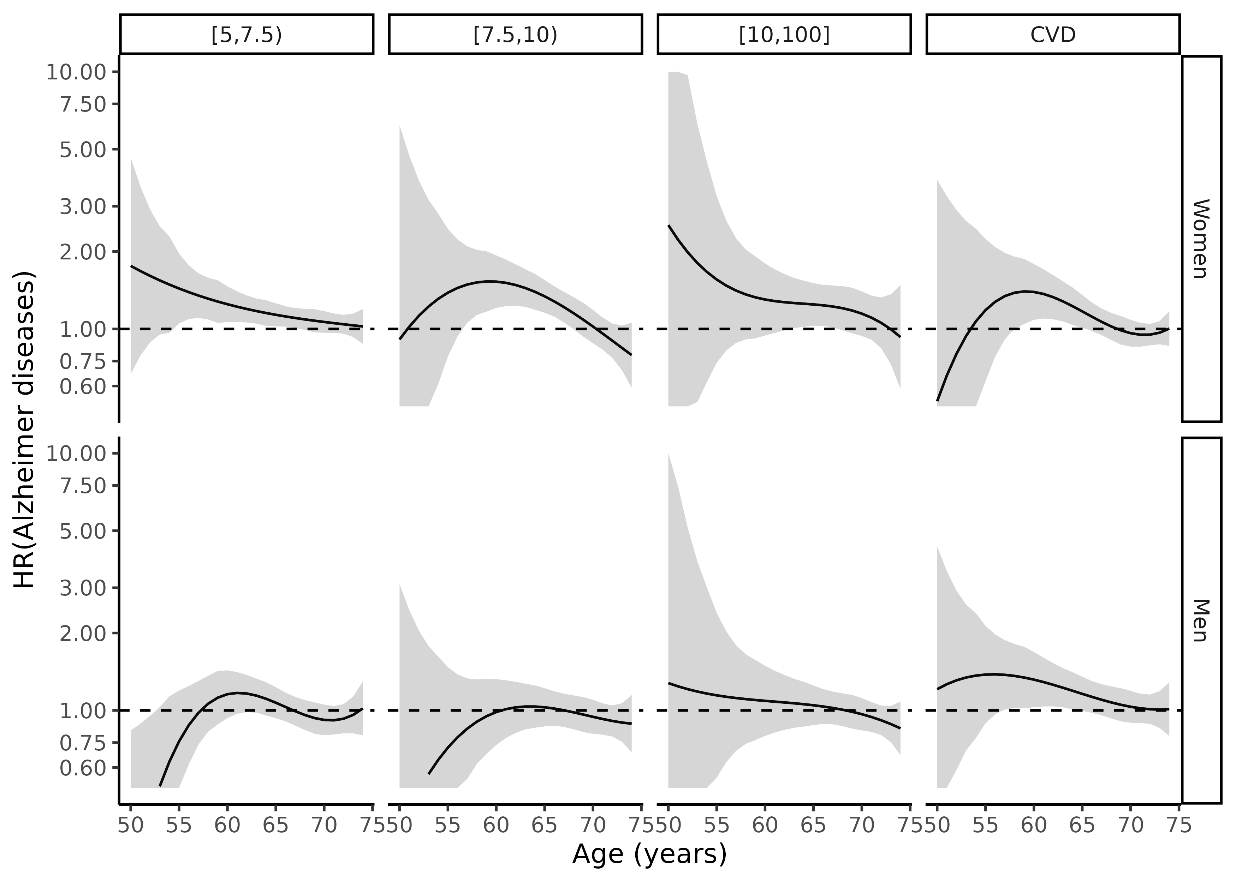
**

**Panel C: Vascular dementia**

**
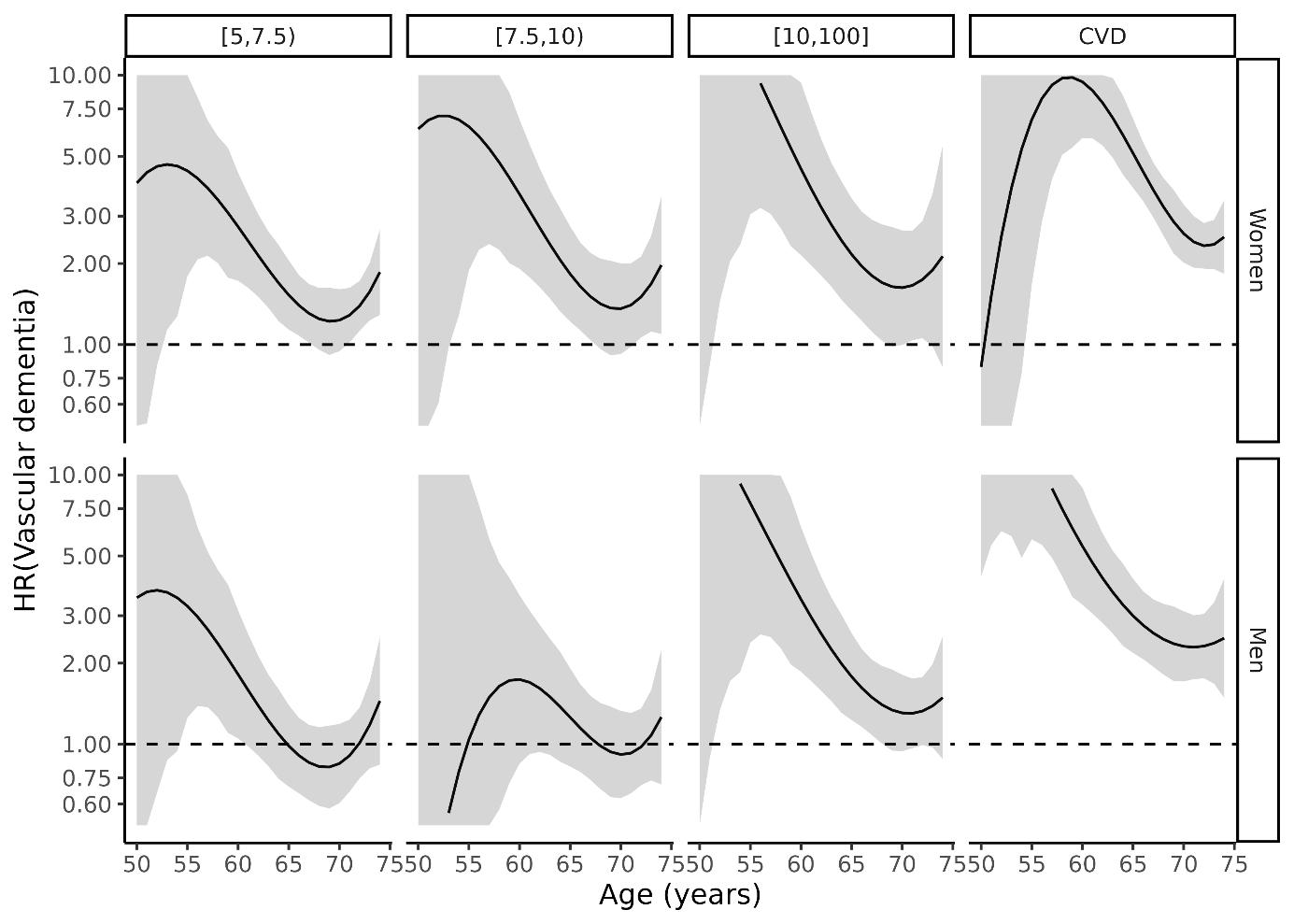
**

**Figure S4.** Cox model b-splines of hazard ratios of dementia types (panel A: all-cause dementia; B: Alzheimer’s disease; C: vascular dementia) according to exposure groups by sex and age. Censuring was applied at the first cardiovascular event or dementia onset during follow-up.

**Panel A: all-cause dementia**

**
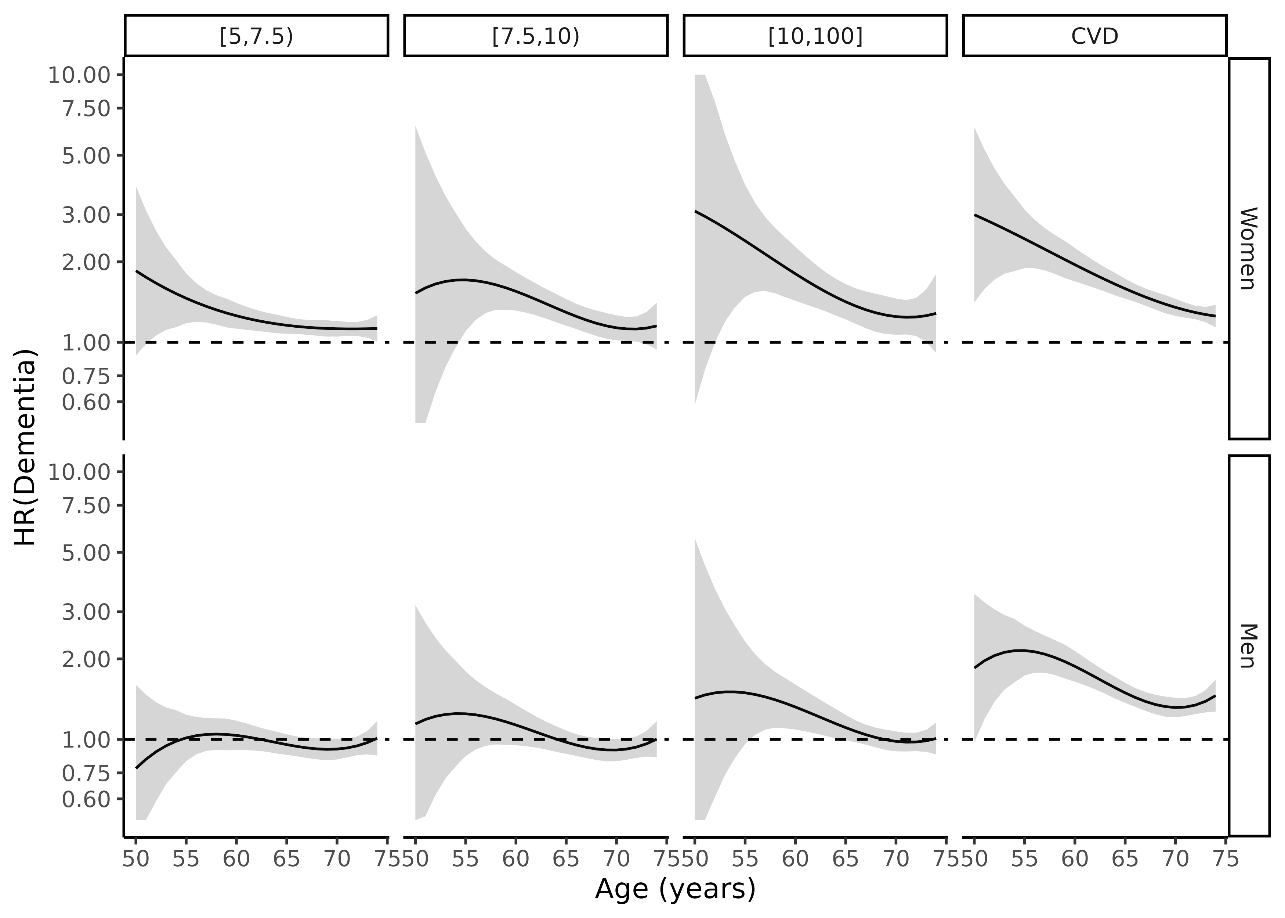
**

**Panel B: Alzheimer’s disease**

**
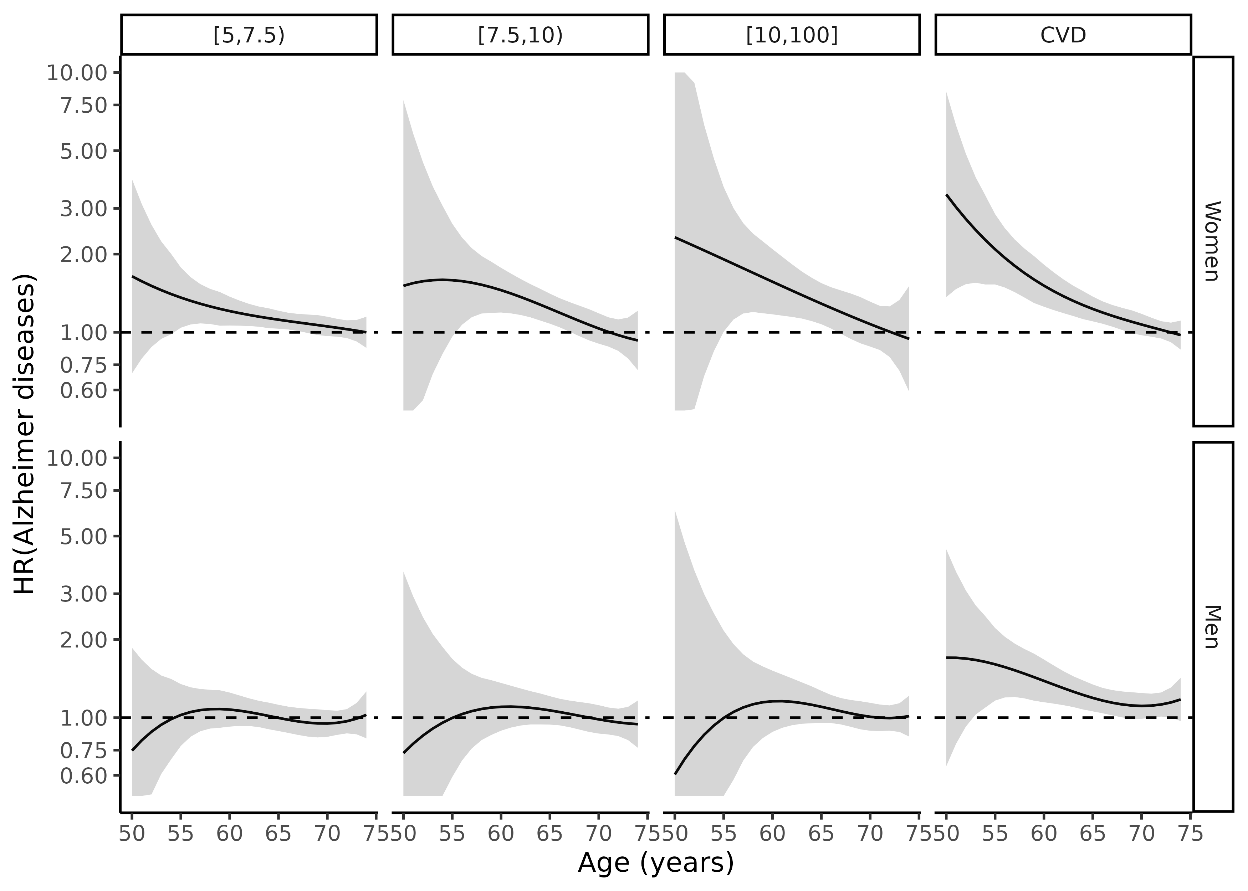
**

**Panel C: Vascular dementia**


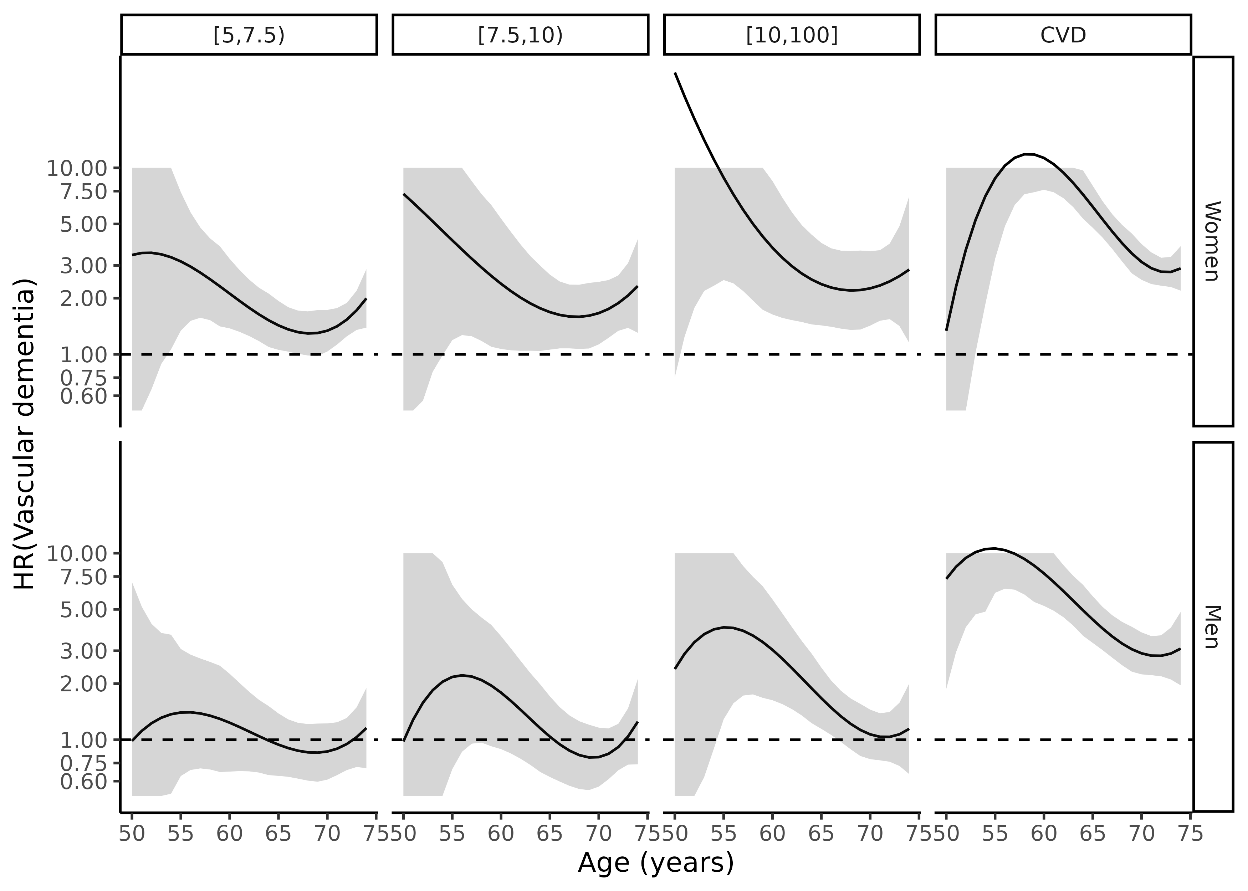

Supplement: Supplementary file 1 — Additional file 1: Box S1. Definition of exclusion criteria. Box S2. Definition of exposure group with previous cardiovascular disease. Box S3. Definition of cases of overall dementia, Alzheimer’s disease, and vascular dementia. Box S4. Definition of baseline covariates. Box S5. Validation of the imputation process. Table S1. Description of missing,complete case and imputed datasets. Table S2. Description of the baseline characteristics of the study population by sex. Table S3. Crude incidences (95% CI) of Alzheimer’s disease by sex, age and exposure groups. Table S4. Crude incidences (95% CI) of vascular dementia by sex, age and exposure groups. Table S5. Subdistribution hazard ratios (95% CI) of the exposure groups obtained in the Fine-Gray models. Figure S1. Flowchart of the study population. Figure S2. Unadjusted Cox model b-splines of hazard ratios of dementia types (panel A: all-cause dementia; B: Alzheimer’s disease; C: vascular dementia) according to exposure groups by sex and age. Figure S3. Complete case analysis to replicate the Cox model b-splines of hazard ratios of dementia types (panel A: all-cause dementia; B: Alzheimer’s disease; C: vascular dementia) according to exposure groups by sex and age. Figure S4. Cox model b-splines of hazard ratios of dementia types (panel A: all-cause dementia; B: Alzheimer’s disease; C: vascular dementia) according to exposure groups by sex and age. Censuring was applied at the first cardiovascular event or dementia onset during follow-up. [file 13195_2024_1406_MOESM1_ESM.docx]
